# Treatment of Diabetes Mellitus Using iPS Cells and Spice Polyphenols

**DOI:** 10.1155/2017/5837804

**Published:** 2017-07-03

**Authors:** Qi Ge, Liang Chen, Keping Chen

**Affiliations:** Institute of Life Science, Jiangsu University, Zhenjiang, Jiangsu 212013, China

## Abstract

Diabetes mellitus is a chronic disease that threatens human health. The disease is caused by a metabolic disorder of the endocrine system, and long-term illness can lead to tissue and organ damage to the cardiovascular, endocrine, nervous, and urinary systems. Currently, the disease prevalence is 11.4%, the treatment rate is 48.2%, and the mortality rate is 2.7% worldwide. Comprehensive and effective control of diabetes, as well as the use of insulin, requires further study to develop additional treatment options. Here, we reviewed the current reprogramming of somatic cells using specific factors to induced pluripotent stem (iPS) cells capable of repairing islet *β* cell damage in diabetes patients to treat patients with type 1 diabetes mellitus. We also discuss the shortcomings associated with clinical use of iPS cells. Additionally, certain polyphenols found in spices might improve glucose homeostasis and insulin resistance in diabetes patients, thereby constituting promising options for the treatment of type 2 diabetes.

## 1. Introduction

Diabetes mellitus (DM) is a hereditary disease caused by the accumulation of glucose in the blood [1]. Studies showed that the number of diabetic patients worldwide exceeded 415 million people by 2015 and is predicted to exceed 642 million by 2040 [2]. DM constitutes a serious chronic noncommunicable disease along with cardiovascular and cerebrovascular diseases and cancer [3]. In both domestic and developed countries, such as Europe and the United States, control and treatment of diabetes is not optimistic. The number of patients diagnosed with diabetes and obesity has increased significantly in recent years [4]. DM leads to islet dysfunction, causing a series of comprehensive metabolic disorders associated with sugars, proteins, fats, or electrolytes [5], and the appearance of high blood sugar causes glycosuria [6]. Although the symptoms of each type of diabetes are generally similar, the causes and population distributions differ. In all types of diabetes, pancreatic *β* cells are unable to produce insulin adequate to lower blood sugar levels, resulting in hyperglycemia [7–9].

## 2. DM Classifications and Pathological Features

DM is produced when the body cannot secrete adequate insulin for its effective use. There are two main forms of DM [9]. Type 1 diabetes, also called insulin-dependent DM, is generally a result of destruction of insulin-producing *β* cells by the immune system [10]. Patients with type 1 diabetes exhibit pancreatic *β* cell damage, resulting in a lack of insulin and ketoacidosis. This may occur at any age but occurs more commonly among younger people. Patients with acute symptoms of metabolic disorders are required to inject insulin. Type 1 DM includes immune-mediated and idiopathic subtypes. Immune-mediated diabetes often involves the presence of one or more autoantibodies, such as islet-cell antibodies, insulin autoantibodies, and glutamate decarboxylase-65 antibodies [11]. Clinical manifestations of type 1 diabetes are as follows: acute onset, disease often due to infection or improper diet, or a family history. Typical symptoms include polyuria, polydipsia, polyphagia, and weight loss. Atypical onset involves patients exhibiting signs of weakness, enuresis, and loss of appetite [12, 13]. The exact mechanism associated with insulin-dependent DM remains unclear; therefore, precautionary measures cannot be taken in advance.

Type 2 diabetes or non-insulin-dependent DM is a common form of insulin resistance that maintains glucose homeostasis by increasing the release of insulin [14]. The etiology of type 2 diabetes was suggested as insulin resistance and inactivation caused by glucotoxicity, lipid toxicity, and inflammation [15]. Glucotoxicity describes a state involving long-term sustainment of high blood glucose levels, and hyperglycemia occurs due to protein glycation [16, 17], which involves a single sugar molecule being covalently bound to the amino group of proteins or the reversible Schiff base of lipids [16]. These reversible Schiff bases are subsequently converted into stable products by intermolecular rearrangement and cross-linking, which results in glycosyl accumulation. Glycosylation plays an important role in structural and functional changes in proteins, which are evident in cases of poorly controlled or uncontrolled DM [18]. Glycation is an unavoidable process during metabolism, and in a hyperglycemic state, the rate of protein glycation and glycosylation increase. Glycosylation products are derived from the cross-linking of structural proteins, which contributes to complications associated with diabetes, including nephropathy, retinopathy, neuropathy, and cardiovascular disease [17].

In addition to type 1 and type 2 diabetes, there is gestational diabetes, as well as other types [19]. Gestational diabetes occurs during the initial stages of pregnancy and is often found in diabetic patients who are pregnant. The pathogenic mechanism associated with gestational diabetes is similar to that of type 2 diabetes, which is also due to insulin resistance. However, unlike type 2 diabetes, insulin resistance in gestational diabetes is a result of hormones secreted by pregnant women [19–22]. The glucose tolerance in some women is restored to normal levels during postpartum periods, whereas others remain at a high risk of diabetes for 5 to 10 years after childbirth [23]. In addition to type I, type II, and gestational diabetes, other special types of diabetes include diabetes caused by pancreatic diseases, endocrine diseases, various genetic abnormalities, and drugs [24]. These types of diabetes, including secondary forms outlined in the 1985 World Health Organization classification criteria, are divided into eight subtypes according to the etiology and pathogenesis [23, 24]. Although there are a number of varieties, the number of patients afflicted with these subsets remains far fewer than those afflicted with type II diabetes [25–27].

## 3. Progress in DM Therapy Research

### 3.1. DM Treatment Using Induced Pluripotent Stem (iPS) Cells

Stem cells possess the unique capability to produce undifferentiated daughter cells or generate specialized cell types when given appropriate signals. The successes of induced formation of *β* cell transplantation opened the door of diabetes therapy [28].  Table 1 briefly summarizes the advantages and disadvantages of current stem cell types used in diabetes research [28–33]. Although there are no currently approved treatments including embryonic stem cells, the regenerative abilities of embryonic stem cells make it ideally suited for autologous grafting of transdifferentiated cells [34]. The current therapies of stem cells provide some theoretical advantages [28, 34]: (1) they are not limited by donor availability; (2) they could provide a long-term source of *β* cells; and (3) they could minimize the need for immunosuppression. Therefore, future research should focus on in vitro expansion of stem cells and the safe reintroduction of these cells into diabetics.

Currently, the clinical treatment of diabetes involves medication and insulin injection. These methods can reduce blood sugar concentration, delay diabetic complications, and improve quality of life; however, they do not constitute a cure [35]. Studies showed that [36] the most important aspect of type 1 DM pathogenesis involved damage to *β* cells, resulting in decreases in their number and the secretion of insulin and causing symptoms related to hyperglycemia. Therefore, increasing the number of *β* cells to restore the function of pancreatic islet cells and the amount of insulin secreted might be a route toward a potential cure. Recently, pancreas and islet-cell transplantation achieved improved clinical outcomes related to diabetes treatment [36]. In 2009, Chambers et al. [37] isolated *β* cell-related islet cells from an adult-donated pancreas and transplanted them into diabetic patients. The experiment was successful, given that the diabetic patients that received the transplants no longer required insulin injections or ingestion of antidiabetic drugs. However, there are deficient resources based on the limited availability of pancreas, pancreatic islet cells, and other donor tissues. Therefore, this method cannot be widely used as a treatment option. Furthermore, the transplanted cells are attacked by the immune system in some patients, although repeat transplantation can often overcome the immune response. Based on these findings, sources of pancreatic *β* cells are the focus of many studies.

**Figure 1:**
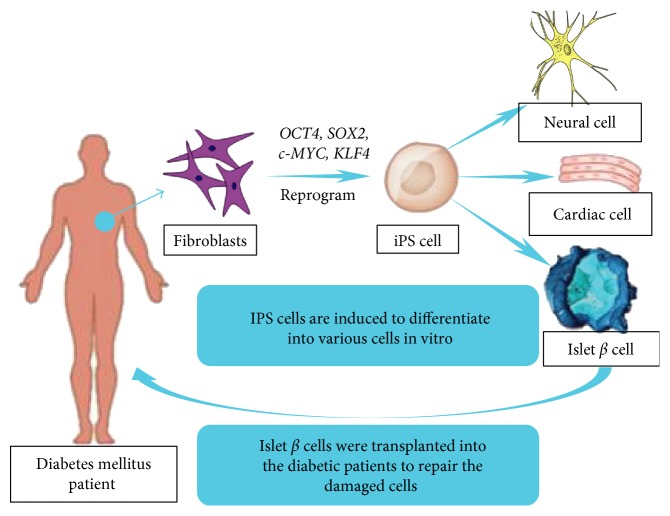
iPS cells induce the formation of pancreatic *β* cells.

Takahashi and Yamanaka [38] revealed the existence of the genes *OCT4*, *SOX2*, *c-MYC*, and *KLF4* related to iPS cells, which led to reports that their expression can lead to the reprogramming of fibroblasts from an adult state into iPS cells (Figure 1). Later experimental results showed that stem cells can reduce blood sugar in type 1 diabetes patients and improve the function of islet cells [39]. Concerning the use of stem cells to treat diabetes, Tateishi et al. [40] successfully isolated insulin-secreting islets by using human embryonic stem cells (ES) and iPS cells to produce insulin-secreting cells using fibroblasts. C-peptide and insulin can be released by glucose stimulation and can be reprogrammed to form iPS cells from somatic cells, after which iPS cells can specifically differentiate into islet *β* cells to offset the lack of pancreatic islet cells in diabetic patients. This suggests that iPS cells have the potential to differentiate into both islet cells and areas of the inner lining of the pancreas similar to human ES cells. However, unlike ES cells, which require specific embryonic tissue to form iPS cells, somatic cells from a patient can be reprogrammed to differentiate into iPS cells [41]. This also enables these cells to avoid triggering the immune response, thereby solving ethical problems and offering a promising diabetes-treatment option.

Tateishi et al. [42] and Park et al. [43] used mouse-skin fibroblasts to reprogram iPS cells, followed by inducing them to differentiate into insulin-secreting cells. The release of insulin increased significantly after injecting these insulin-secreting cells into the portal vein of diabetic mice. Additionally, data indicated that blood glucose levels decreased, and the level of HbA1c also reverted to levels close to those observed in nondiabetic patients. These results showed that iPS cells could be used successfully for the treatment of islet-cell damage in diabetic mice. In 2008, Park et al. [43] established a variety of iPS cell lines, including those derived from patients with type 1 diabetes, and Zhang et al. [44] and Maehr [45] successfully differentiated human iPS cells into insulin-secreting cells. These results showed that iPS cells were capable of addressing decreased amounts of islet cells in diabetic patients to overcome limitations in lace concerning the lack of donor tissue, as well as immune rejection experienced following islet-transplantation therapy, thereby offering new hope for diabetic patients.

The discovery of human pluripotent stem cells (hPSC) opened the possibility of generating replacement cells and tissues in the laboratory that could be used for diabetes treatment and drug screening [45]. Pagliuca et al.'s [46] research showed that the generation of insulin-producing pancreatic *β* cells from stem cells in vitro would provide an unprecedented cell source for drug discovery and cell transplantation therapy in diabetes. They reported a scalable differentiation protocol that can generate hundreds of millions of glucose-responsive *β* cells from hPSC in vitro. These stem-cell-derived *β* cells (SC-*β*) express markers found in mature *β* cells and flux Ca^2+^ in response to glucose. *β* cells sense the changing glucose levels through calcium signaling, and increasing glucose levels leads to membrane depolarization, causing an influx of calcium ions, which triggers insulin exocytosis [46, 47]. In addition, these cells secrete human insulin into the serum of mice after transplantation in a glucose-regulated manner, and transplantation of these cells improves hyperglycemia in diabetic mice. And then, by using sequential modulation of multiple signaling pathways in a three-dimensional cell culture system, without any transgenes or genetic modification, they generated glucose-responsive cells that show key features of *β* cells both in vitro and in vivo. It also shows that the potential utility of these cells for transplantation therapy for diabetes in vivo. Furthermore, with continued research, iPS cells and other stem-cell-based therapies have the potential to move medicine toward a permanent cure for type 1 diabetes [28, 29].

Thus far, there is no other effective method of reversing autoimmunity once a patient enters the course of type 1 diabetes without cell transplantation therapy. The researches show that the regeneration of pancreatic islets are ultimate goals for the complete cure of type 1 diabetes [30]. Herein, we reviewed the therapeutic effects of iPS cells on type 1 diabetes. However, several clinical trials of spice-diet therapy in diabetes mellitus patients aimed at preventing or delaying disease progression [31]. This combination with cell therapy will be a new approach of treating diabetes mellitus.

### 3.2. DM Treatment Using Spice Polyphenols

**Figure 2:**
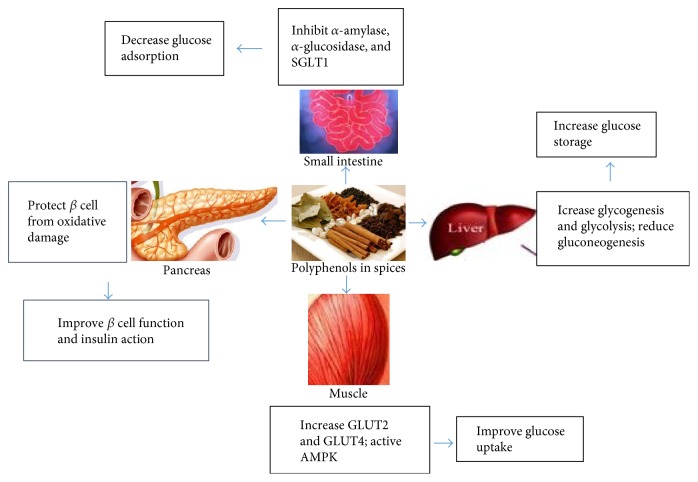
The beneficial effects of spice polyphenols on glucose homeostasis and insulin resistance.

Some medications taken for diabetes treatment exhibit toxic side effects, with long-term exposure to some medications also weakening the response to their effects. For example, metformin hydrochloride tablets can cause gastrointestinal discomfort. However, phenolic compounds found in edible plants have attracted increasing attention due to their efficacy for the prevention of diabetes. Compared with synthetic drugs, edible portions of plants are natural, economic, and environmentally safe. In addition to fruits and vegetables, spices are the main sources of dietary phenolic compounds, with polyphenols found in ~80 spices exhibiting antisugar effects related to the prevention and control of diabetes [48]. Phenols and polyphenols might participate in glucose-metabolism pathways [48–50] related to the absorption of glucose in the intestine, insulin secretion by islet *β* cells, regulation of glucose production in the liver, insulin-receptor activity in insulin-sensitive tissues and glucose uptake, and regulation of intrahepatic glucose output (Figure 2). Therefore, the discovery of these compounds in seasonal foods might not only enhance their antioxidant effects but also exert antisugar effects.

#### 3.2.1. The Effect of Spice Compounds on DM

Many of the positive health effects of spice compounds are attributed to phenolic compounds. These compounds include polyphenols, terpenoids, vanilla, and organic sulfur in common spices (Table 2) [50]. Polyphenols are classified as flavonoids, including flavanones, flavones, and flavonols, and nonflavonoids, such as phenolic acids. Flavonoids exhibit antioxidant, anticancer, antiallergy, anti-inflammatory, and protective effects against gastric ulcers. Recent studies [51] showed that terpenoids, vanilla, and organic sulfur compounds also exhibit antioxidative properties and aid in the prevention of chronic diseases, such as diabetes.

Various spices and spice compounds (Table 2) have been successfully applied for the regulation of type 2 diabetes, which accounts for ~90% of DM cases [48–54]. Although the beneficial effects of spice compounds can reduce fasting and postprandial blood glucose levels, their mechanisms of action remain difficult to understand, given that different spices contain a variety of phenolic compounds that may act synergistically [54]. Therefore, further studies are necessary to gain a better understanding of the antidiabetic potential of biologically active compounds present in spices to increase their utilization in helping prevent diabetes, complications of diabetes, and metabolic abnormalities. Furthermore, the beneficial effects and bioactivity of other common spices, including cinnamon, ginger, turmeric, cumin, fenugreek, garlic and onions, cloves, black pepper, and curry, have also been evaluated [55–61] for their potential use in DM management (Figure 2).

#### 3.2.2. The Hypoglycemic Effects of Cinnamon

Although a variety of spices are used to enhance flavor, some exhibit side effects related to reducing blood sugar levels according to clinical trials involving animals and humans [48]. Cinnamon is the most frequently consumed spice in the world [62] and has been granted GRAS (Generally Recognized As Safe) classification by the United States Food and Drug Administration [63]. Many studies confirmed that cinnamon is rich in cinnamaldehydes A and B, which are the sources of the antioxidant, anti-inflammatory, antibacterial, antiulcer effects. Khan et al. [64] showed that cinnamon contains an islet-enhancing factor potentially involved in relieving diabetes-related symptoms and other insulin-related issues. Other spices in the *Cinnamomum* genus, including camphor and ceylon cinnamon, were also identified as being capable of improving responses to increased blood glucose levels. Among these, Chinese cinnamon exhibited the most favorable profile for treating hyperglycemia in type 2 diabetes patients [63] through mechanisms involved in stimulating the secretion of insulin and insulin analogues, increasing the expression of glucagon-like peptide-1 (GLP-1), delaying gastric emptying, inhibiting glucosidase activity, and increasing the expression of glucose transporter-4 [65].

In vitro and in vivo studies reported antidiabetes properties associated with cinnamon. Imparl-Radosevich et al. [66] showed that polyphenol compounds extracted from cinnamon exhibited insulin-like properties in vitro capable of inhibiting the activity of protein tyrosine phosphatase or serine phosphorylation of insulin-receptor substrate 1. Based on these findings, it was suggested that cinnamon might be useful for the treatment of DM involving insulin resistance and metabolic syndrome. Compared with the activity of insulin or insulin analogues, 49 common herbs, spices, and medicinal plants were extracted to determine their in vitro effects on mouse epididymal fat cells. The results indicated that cinnamon extract enhanced the insulin activity by 20-fold relative to the effects of other spices and herbal extracts [67].

Additionally, the insulin-enhancing effects of cinnamon were also reported in animal and human trials. Qin et al. [68] observed that administration of cinnamon extract improved glucose utilization in normal rats after ingestion of foods containing high concentrations of fructose. Additionally, cinnamon extract enhances the effect of insulin and improves glucose metabolism; mice injected with cinnamon extract exhibited higher glucose-injection volumes as compared with controls [62]. Cinnamon is also effective at increasing high-density lipoprotein levels in diabetic mice by lowering blood glucose, total cholesterol, and triglyceride levels [69]. The antidiabetic and hypolipidemic effects of cinnamon might be due to cinnamaldehyde [70], the administration of which significantly decreased plasma glucose, total cholesterol, and triglyceride levels in streptozotocin-treated diabetic rats. Another study [71] reported that cinnamon oil or extracts rich in polyphenol oligomers decreased rates of hypoglycemia and exhibited antioxidant effects in diabetic rats. Furthermore, cinnamon polyphenols exhibit insulin-like and insulin-independent activity regulating gene expression and alter insulin-signaling pathways in mouse adipocytes [72].

In 2003, Khan et al. [73] conducted a random, double-blind controlled clinical trial to assess the effects of cinnamon in patients with type 2 diabetes. Sixty patients (30 men and 30 women) received placebos or three different doses of cinnamon powder (1, 3, and 6 g/day) for 40 days, with results indicating that cinnamon intake reduced fasting blood glucose levels. Studies also showed that cinnamon can reduce triglyceride, low-density lipoprotein, and total cholesterol levels in diabetic patients [73]. The effect of cinnamon on blood glucose and blood lipid levels might be due to its ability to increase glycogen synthase activity, increase the uptake of glucose, and inhibit glycogen synthase kinase 3*β* and dephosphorylation of insulin receptors [74]. However, the effects of cinnamon used for the treatment of type 2 diabetes differs from person to person. Blevins et al. [75] did not observe a significant improvement in glycosylated hemoglobin in 43 diabetes patients administered cinnamon.

Lu et al. [76] showed that application of cinnamon extract resulted in a dose-dependent decrease in fasting plasma glucose and glycosylated hemoglobin levels. Participants in that study exhibited similar glycosylated hemoglobin levels during the early stages of experiments and had different fasting blood glucose levels, which represented confounding factors for their results. Additionally, it remained uncertain whether placebos showed any observable effects due to the relatively low initial fasting blood glucose levels measured in patients. Although it was suggested that cinnamon could help reduce glycosylated hemoglobin and fasting blood glucose levels in diabetic patients, the mechanism of action remains unknown. In this study by Lu et al. [76], both placebo and treatment resulted in similar initial fasting glucose levels, which subsequently decreased, whereas another study [77] showed that administration of 2 g of cinnamon reduced glycosylated hemoglobin and blood glucose levels in type 2 DM patients.

Mang et al. [78] studied the effects of cinnamon extract on plasma glucose, glycohemoglobin, and blood lipid levels in type 2 DM patients, with their results showing that cinnamon extract exhibited a significant effect on reducing glycosylated hemoglobin levels in diabetic patients with poor blood glucose levels (Table 3). Additionally, Crawford [79] reported that cinnamon reduced glycosylated hemoglobin levels in 109 type 2 diabetes patients. These results suggested that cinnamon in its various forms has the potential to lower diabetes-related indicators in the absence of side effects. Therefore, cinnamon can assist in the treatment of type 2 diabetes; however, further research is needed to confirm the mechanisms associated with the antidiabetes effects of cinnamon.

## 4. New Approach in Diabetes Therapy

Overcoming diabetes is a long-standing problem. A variety of hypoglycemic drugs and drug targets, including sulfonylureas, biguanides, *α*-glucosidase inhibitors, nonsulfonylurea drugs, thiazolidinediones, GLP-1-receptor agonists, dipeptidyl peptidase-4 inhibitors, and sodium-glucose cotransporter-2 inhibitors, have been discovered to address the pathogenesis of diabetes [80]. The mechanisms of antidiabetic drugs include (1) stimulating insulin release by inhibiting the adenosine triphosphate-sensitive potassium (KATP) channel [81], (2) reducing gluconeogenesis and increasing 5′ adenosine monophosphate-activated protein kinase signaling to reduce insulin resistance [82], (3) mitigating insulin resistance by activating peroxisome proliferator-activated receptor gamma in fat and muscle [83], and (4) reducing the absorption of glucose by the small intestine [84]. In addition to these drugs, new therapies for diabetes are continuously being developed.

A recent study by Toda et al. [85] showed that mitochondria in brain neurons play a crucial role in systemic glycemic control. Their results indicated that increases in blood sugar levels led to morphological changes in neuronal mitochondria, thereby altering their function. This mechanism might be important for the development of metabolic diseases, such as type 2 diabetes. Dooley et al. [86] reported that genetic defects in *β* cells were common between type 1 and type 2 diabetes, findings that some genes important to *β* cell survival can be used to distinguish between diabetic phenotype based on the ability of *β* cells to withstand external stress. Additionally, Scarlett et al. [87] utilized injections of fibroblast growth factor-1 into the ventricles of mice with type 2 diabetes to successfully lower blood sugar levels, with the efficacy of this treatment lasting up to 18 weeks and accompanied by normalized blood glucose levels. A study by Bader et al. [88] revealed that the protein marker Flattop, present in 80% of *β* cells, could subdivide insulin-producing pancreatic *β* cells into two categories. One set can effectively determine the concentration of glucose in the environment and secrete the necessary amount of insulin, thereby indicating metabolic properties of mature *β* cells. By contrast, *β* cells lacking Flattop exhibited a particularly high rate of proliferation and represented immature reserves constantly renewing themselves to replenish mature *β* cells. Separation of the two subtypes is expected to promote analysis of relevant signaling pathways and aid the development of options related to regenerative therapy. Inflammation can induce heart disease, stroke, kidney disease, and other related complications in diabetes patients. Wei et al. [89] identified chronic inflammation as a possible mechanism for triggering diabetes, showing that deletion of fatty acid synthase in macrophages prevents diet-induced insulin resistance, recruitment of macrophages to adipose tissue, and chronic inflammation. Another study by Li et al. [90] reported new pathogenic pathways and drug targets for type 2 diabetes. This study showed that Galectin-3 (Gal3), an inflammatory cytokine secreted by macrophages, can bind to insulin receptors and interfere with related signaling pathways, resulting in insulin resistance. Additionally, their results found that significantly elevated blood Gal3 levels in obese patients were positively correlated with homeostatic model assessment—insulin-resistance index values—and that Gal3 also induced insulin resistance in human muscle cells. These results indicated that Gal3 was capable of inducing insulin resistance in obese patients. Subsequent studies on Gal3 inhibition showed that Gal3 knockout or administration of a Gal3 inhibitor significantly improved levels of insulin resistance in obese mice, suggesting Gal3 as a potential drug target related to treatment of insulin resistance and diabetes.

## 5. Summary and Prospects

These findings described here suggested that iPS cells and spices could potentially serve as a therapeutic modality for diabetes mellitus. Apart from this, the studies also showed that stable polyphenol compounds in spices could enhance insulin secretion and confer strong resistance to *β* cell destruction. Therefore, use of combination high-dose spices and iPS cell therapy was well tolerated and may have beneficial effects on *β* cells function. Although we cannot establish a stable association between iPS cell therapy and spice-diet, the results observed in this aspect are encouraging showing improvement of *β* cell mass and function in diabetes mellitus [30, 91]. These studies indicated that iPS cell therapy and spice-diet can have a strong influence on pancreatic islet function and immune response.

The establishment of iPS cells and related research has brought hope for improvements in the treatment of diabetes. iPS-cell-related mechanisms and their applications offer potential for development of a new field of regenerative medicine. Although there have been many successes in this field of research, multiple key issues remain, including the appropriate method of applying the technology for diabetes treatment. These involve improving methods related to the induction efficiency of iPS cells, solving problems of directional differentiation, controlling the safety of clinical treatment, reducing the tumorigenicity of iPS cells, and ensuring that iPS cells can be transplanted free of side effects. To this end, it is important to select highly differentiated pancreatic cells for induction to specific *β* cells.

Spices exhibit beneficial effects to human health and may constitute better prospects for therapeutic use than the unidirectional differentiation of cells. Currently, there are recommendations for the daily consumption of edible spices rich in bioactive ingredients. However, consumers should consume spices with caution due to their potentially adverse effects over the long term. Scientific evidence related to the health benefits associated with spices will be expanded upon in future work.

Diabetes is a global epidemic that presents a major challenge in the regulation of its complications. Understanding the pathogenic pathways related to diabetes contributes to the successful development of treatment options. Spices are natural products rich in high concentrations of antioxidant compounds, and their potential antidiabetic effects, including anti-inflammatory and antihyperglycemic activity, are well studied. The use of spice compounds has the potential to aid in the treatment of diabetes and limit its associated complications, as well as forming a combined treatment through the use of synthetic spices. Seasonal spiced foods may allow for increases in the daily absorption of antioxidants and provide a means of reducing risks associated with the treatment of diabetes and metabolic abnormalities. Although clinical trials have been undertaken to assess the effect of spice compounds on diabetes treatment, there remains insufficient evidence to definitively determine their effectiveness. Additionally, spices are often used in small amounts, with their compounds and antioxidant activities easily affected during the food production process, thereby limiting their therapeutic potential. In the future, further clinical research will be required to confirm the efficacy of spice compounds for use in the treatment and/or prevention of diabetes.

## Figures and Tables

**Table 1:** Summary of stem cell-based therapies.

Types of stem cells	Advantages	Disadvantages	References
iPS cells	Avoiding ethical question and immune rejection; potentially unlimited supply	Have carcinogenic effects	[28, 29]
Pancreatic stem cells	Partially differentiated toward *β* cells	Difficulty isolating cells and transdifferentiation factors	[28, 29, 32]
Hepatic stem cells	Ideal autologous source; endodermal origin	Difficulty achieving sufficient in vitro cell mass for use in transplant therapy	[28, 33]
Embryonic stem cells	Potentially unlimited supply; self-renewal and multi-directional differentiation	Ethical constraints; the possibility of forming teratomas; cause autoimmune response	[28, 29]
Spermatogonial stem cells	Potentially unlimited supply; avoiding ethical question; unlimited plasticity	No long-term studies; male centric	[28, 92]
Mesenchymal stem cells	Potentially unlimited supply; with multidirectional differentiation potential; autologous transplantation	Require chronic administration and adjunct therapy; effects are incomplete and temporary; their potential immunotolerance and anti-inflammatory properties in vivo are not clear	[28, 52]

**Table 2:** The effective role of spices in the control of type 2 diabetes mellitus.

Spices	Picture	Active compound	Test methods	Beneficial effects	References
Cinnamon	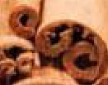	Cinnamaldehyde	Randomized double-blind test, parallel control experiment, capsule dose of 1, 3, and 6 g/day	Cinnamon decreased plasma glucose, total cholesterol, LDL cholesterol levels	[45]
Ginger	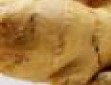	Enone, honeydone	Randomized double-blind test, parallel control experiment, capsule dose 3 g/day	Ginger to reduce the body of FBG, HbA1c also improve insulin resistance	[15, 48]
Turmeric	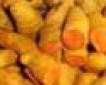	Curcumin	Standard metformin and supplemented with 2 g of turmeric	Curcuma is useful on blood sugar, oxidative stress, inflammation	[15]
Cumin	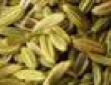	Anisole alcohol	The dose of black fennel is 1, 2, and 3 g/day	Daily fennel 2 g can significantly reduce blood glucose levels	[50]
Coriander	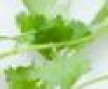	Phenols, flavonoids	Coriander seed powder dose 5 g/day	Coriander and anise seeds can reduce FBG, plasma lipids, lipoproteins	[51, 53]
Anise	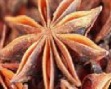	Anethole	Octagonal powder dose 5 g/day	Improvement of HDL control of plasma lipid peroxidation	[49]
Fenugreek	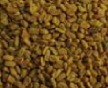	Saponin	2.5 g of fenugreek leaves were mixed with water	Fenugreek lowers blood sugar levels and glycerol triphosphate	[44]
Onion	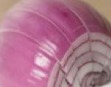	Flavonoids	Daily doses of 25, 50, 75, and 100 g of fresh onion slices	Onion intake can reduce FBG levels	[54]
Clove	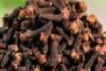	Eugenol	Daily doses were 0, 1, 2, and 3 g	Reduce serum glucose, triglycerides, total cholesterol, LDL	[93]

**Table 3:** The effective control of spices extracts in type 2 diabetes mellitus.

Spices extracts	Preparation methods	Experimental model	Effect	References
Cinnamon extract	112 mg of the aqueous cinnamon extract was prepared from 1 g of cinnamon	Type 2 diabetes patients	Lower fasting blood glucose	[94]
Turmeric extract	Turmeric powder is a semisolid material obtained from ethanol and evaporated, and the extract contains an oleaginous resin in an amount of between 75% and 85%	Type 2 diabetes patients	Improve the islet *β* cell function	[95]
Garlic powder tablets	The garlic powder tablet contains 150 mg of dehydrated garlic powder	Type 2 diabetes patients	Reduce FBG, triglycerides	[96]

## References

[1] Shaw J. E., Sicree R. A., Zimmet P. Z. (2010). Global estimates of the prevalence of diabetes for 2010 and 2030. *Diabetes Research & Clinical Practice*.

[2] Michels A. W., Eisenbarth G. S. (2011). Immune intervention in type 1 diabetes. *Seminars in Immunology*.

[3] Kwak S. H., Park K. S. (2016). Recent progress in genetic and epigenetic research on type 2 diabetes. *Experimental & Molecular Medicine*.

[4] Li Z., Eisenbarth G. S. (2011). Prediction and prevention of type 1 diabetes mellitus. *Journal of Diabetes*.

[5] Rao A. A., Sridhar G. R., Srinivas B., Das U. N. (2008). Bioinformatics analysis of functional protein sequences reveals a role for brain-derived neurotrophic factor in obesity and type 2 diabetes mellitus. *Medical Hypotheses*.

[6] Chadban S., Howell M., Twigg S. (2010). The CARI guidelines. Prevention and management of chronic kidney disease in type 2 diabetes. *Nephrology*.

[7] Srikanth S., Deedwania P. (2011). Primary and secondary prevention strategy for cardiovascular disease in diabetes mellitus. *Cardiology Clinics*.

[8] Anderson B. J. (2004). Family conflict and diabetes management in youth: clinical lessons from child development and diabetes research. *Diabetes Spectrum*.

[9] West S. D., Nicoll D. J., Wallace T. M., Matthews D. R., Stradling J. R. (2007). Effect of CPAP on insulin resistance and HbA1c in men with obstructive sleep apnoea and type 2 diabetes. *Thorax*.

[10] Franciosi M., Pellegrini F., De Berardis G. (2001). The impact of blood glucose self-monitoring on metabolic control and quality of life in type 2 diabetic patients: an urgent need for better educational strategies. *Diabetes Care*.

[11] Xie C., Liao R. (2014). Research progress of the model of diabetes mellitus health education. *Chinese Medicine Modern Distance Education of China*.

[12] Nie M., Zhou X., Liu D. (2016). Study on the treatment of diabetic foot with Chinese medicine. *China Continuing Medical Education*.

[13] Meng Q. Y., Jian-Wei M. A., Dong J., Wei H. L., Ma X. Y., Zhi Y. (2015). A study on characteristics of TCM syndrome factors and syndrome differentiation types of type 2 diabetes mellitus patients with dyslipidemia by factor analysis and cluster analysis. *Medical & Pharmaceutical Journal of Chinese Peoples Liberation Army*.

[14] Chehade J. M., Mooradian A. D. (2000). A rational approach to drug therapy of type 2 diabetes mellitus. *Drugs*.

[15] Selvi N. M. K., Sridhar M. G., Swaminathan R. P., Sripradha R. (2015). Efficacy of turmeric as adjuvant therapy in type 2 diabetic patients. *Indian Journal of Clinical Biochemistry*.

[16] Maithili K. S. N., Sridhar M. G., Swaminathan R. P., Sripradha R. (2015). Efficacy of turmeric as adjuvant therapy in type 2 diabetic patients. *Indian Journal of Clinical Biochemistry*.

[17] Kim S. Y., England J. L., Sharma J. A., Njoroge T. (2011). Gestational diabetes mellitus and risk of childhood overweight and obesity in offspring: a systematic review. *Experimental Diabetes Research*.

[18] Mang B., Wolters M., Schmitt B. (2006). Effects of a cinnamon extract on plasma glucose, HbA 1c, and serum lipids in diabetes mellitus type 2. *European Journal of Clinical Investigation*.

[19] Prior J. O., Quiñones M. J., Hernandez-Pampaloni M. (2005). Coronary circulatory dysfunction in insulin resistance, impaired glucose tolerance, and type 2 diabetes mellitus. *Circulation*.

[20] Xie C., Liao R. (2014). Research progress of the model of diabetes mellites health education. *Chinese Medicine Modern Distance Education of China*.

[21] Zhou W., Li H., Fang Z. (2016). Present situation and prospect of community diabetes health education. *Health Education & Health Promotion*.

[22] Yan-Hong L. I., Shan Y. (2009). Research progress of health education in diabetic patients. *Journal of Nursing Administration*.

[23] Muoio D. M., Newgard C. B. (2008). Mechanisms of disease: Molecular and metabolic mechanisms of insulin resistance and beta-cell failure in type 2 diabetes. *Nature Reviews Molecular Cell Biology*.

[24] Wang M. J., Yan Z. L. (2014). Research progress on the application of proteomics in obesity, insulin resistance and type 2 diabetes. *Chinese General Practice*.

[25] Organization. W H (1999). Definition, diagnosis and classification of diabetes mellitus and its complications: report of a WHO consultation. *Journal of Medical Genetics*.

[26] Association A D (1998). Diagnosis and classification of diabetes mellitus. *American Family Physician*.

[27] Nathan D., Buse J. M., Ferrannini E., Holman R. R., Sherwin R., Zinman B. (2009). Management of hyperglycaemia in type 2 diabetes mellitus: a consensus algorithm for the initiation and adjustment of therapy: update regarding the thiazolidinediones. *Diabetologia*.

[28] Godfrey K. J., Mathew B., Bulman J. C., Shah O., Clement S., Gallicano G. I. (2012). Stem cell-based treatments for type 1 diabetes mellitus: bone marrow, embryonic, hepatic, pancreatic and induced pluripotent stem cells. *Diabetic Medicine*.

[29] Lilly M. A., Davis M. F., Fabie J. E., Terhune E. B., Gallicano G. I. (2016). Current stem cell based therapies in diabetes. *American Journal of Stem Cells*.

[30] Bi X., Li F., Liu S. (2017). *ω*-3 polyunsaturated fatty acids ameliorate type 1 diabetes and autoimmunity. *Journal of Clinical Investigation*.

[31] Baidal D. A., Ricordi C., Garcia-Contreras M., Sonnino A., Fabbri A. (2016). Combination high-dose omega-3 fatty acids and high-dose cholecalciferol in new onset type 1 diabetes: a potential role in preservation of beta-cell mass. *European Review for Medical & Pharmacological Sciences*.

[32] Kahan B. W., Jacobson L. M., Hullett D. A. (2003). Pancreatic precursors and differentiated islet cell types from murine embryonic stem cells: an in vitro model to study islet differentiation. *Diabetes*.

[33] Jiao F., Hu H., Han T. (2015). Long noncoding RNA MALAT-1 enhances stem cell-like phenotypes in pancreatic cancer cells. *International Journal of Molecular Sciences*.

[34] McCall M. D., Toso C., Baetge E. E., Shapiro A. M. (2010). Are stem cells a cure for diabetes?. *Clinical Science*.

[35] Drago R., Wooden M. (2007). Treatment of sickle cell anemia mouse model with iPS cells generated from autologous skin. *Science*.

[36] Zhang D., Jiang W., Liu M. (2009). Highly efficient differentiation of human ES cells and iPS cells into mature pancreatic insulin-producing cells. *Cell Research*.

[37] Chambers S. M., Fasano C. A., Papapetrou E. P., Tomishima M., Sadelain M., Studer L. (2009). Highly efficient neural conversion of human ES and iPS cells by dual inhibition of SMAD signaling. *Nature Biotechnology*.

[38] Takahashi K., Yamanaka S. (2006). Induction of pluripotent stem cells from mouse embryonic and adult fibroblast cultures by defined factors. *Cell*.

[39] Takahashi K., Tanabe K., Ohnuki M. (2007). Induction of pluripotent stem cells from adult human fibroblasts by defined factors. *Cell*.

[40] Tateishi K., He J., Taranova O., Liang G., D’Alessio A. C., Zhang Y. (2008). Generation of insulin-secreting islet-like clusters from human skin fibroblasts. *Journal of Biological Chemistry*.

[41] Stojanovic I. (2009). *IPS Cells from Type I Diabetes Mellitus for Disease Modeling and Therapy - a Review on the Possibilities and Limitations*.

[42] Tateishi K., He J., Taranova O., Liang G., D’Alessio A. C., Zhang Y. (2008). Generation of insulin-secreting islet-like clusters from human skin fibroblasts. *Journal of Biological Chemistry*.

[43] Park I. H., Arora N., Huo H. (2008). Disease-specific induced pluripotent stem (iPS) cells. *Cell*.

[44] Zhang D., Jiang W., Liu M. (2009). Highly efficient differentiation of human ES cells and iPS cells into mature pancreatic insulin-producing cells. *Cell Research*.

[45] Maehr R. (2011). iPS cells in type 1 diabetes research and treatment. *Clinical Pharmacology & Therapeutics*.

[46] Pagliuca F. W., Millman J. R., Gürtler M. (2014). Generation of functional human pancreatic *β* cells in vitro. *Cell*.

[47] Mohammed J. S., Wang Y., Harvat T. A., Oberholzer J., Eddington D. T. (2008). Microfluidic device for multimodal characterization of pancreatic islets. *Lab on a Chip*.

[48] Mozaffari-Khosravi H., Talaei B., Jalali B. A., Najarzadeh A., Mozayan M. R. (2014). The effect of ginger powder supplementation on insulin resistance and glycemic indices in patients with type 2 diabetes: a randomized, double-blind, placebo-controlled trial. *Complementary Therapies in Medicine*.

[49] Maithili K. S. N., Sridhar M. G., Swaminathan R. P., Sripradha R. (2015). Efficacy of turmeric as adjuvant therapy in type 2 diabetic patients. *Indian Journal of Clinical Biochemistry*.

[50] Bamosa A. O., Kaatabi H., Lebdaa F. M., Elq A. M., Al-Sultanb A. (2010). Effect of *Nigella sativa* seeds on the glycemic control of patients with type 2 diabetes mellitus. *Indian Journal of Physiology & Pharmacology*.

[51] Jamshidzadeh A., Heidari R., Razmjou M. (2015). An in vivo and in vitro investigation on hepatoprotective effects of *Pimpinella anisum* seed essential oil and extracts against carbon tetrachloride-induced toxicity. *Iranian Journal of Basic Medical Sciences*.

[52] Sánchez L., Gutierrez-Aranda I., Ligero G. (2015). Enrichment of human ESC-derived multipotent mesenchymal stem cells with immunosuppressive and anti-inflammatory properties capable to protect against experimental inflammatory bowel disease. *Stem Cells*.

[53] Rajeshwari U., Shobha I., Andallu B. (2011). Comparison of aniseeds and coriander seeds for antidiabetic, hypolipidemic and antioxidant activities. *Spatula DD-Peer Reviewed Journal on Complementary Medicine and Drug Discovery*.

[54] Mitra A., Bhattacharya D., Roy B. C. (2006). Dose-dependent effects of fenugreek composite in diabetes with dyslipidemia. *Internet Journal of Food Safety*.

[55] Goswami P., Mandal P., Jha P., Misra T., Barat S. (2013). Antioxidant activities of different spices on the lipid oxidation of cooked and uncooked fillet of two fish species belonging to the genus Puntius. *Journal of Agricultural Science & Technology*.

[56] Guan F., Ding Y., Zhang Y., Zhou Y., Li M., Wang C. (2016). Curcumin suppresses proliferation and migration of MDA-MB-231 breast cancer cells through autophagy-dependent Akt degradation. *PLoS One*.

[57] Kunnumakkara A. B., Bordoloi D., Padmavathi G. (2016). Curcumin, the golden nutraceutical: multitargeting for multiple chronic diseases. *British Journal of Pharmacology*.

[58] Park J. H. (2016). Interactive effects of fenugreek (*Trigonella foenum-graecum* L.) seed extract supplementation and dietary metabolisable energy levels on the growth performance, total tract digestibility, blood profiles, and excreta gas emission in broiler chickens. *Animal Production Science*.

[59] Banerji S., Banerjee S. (2016). A formulation of grape seed, Indian gooseberry, turmeric and fenugreek helps controlling type 2 diabetes mellitus in advanced-stage patients. *European Journal of Integrative Medicine*.

[60] Ibrahim Z. S., Alkafafy M. E., Ahmed M. M., Soliman M. M. (2016). Renoprotective effect of curcumin against the combined oxidative stress of diabetes and nicotine in rats. *Molecular Medicine Reports*.

[61] Guo S., Long M., Li X., Zhu S., Zhang M., Yang Z. (2016). Curcumin activates autophagy and attenuates oxidative damage in EA.hy926 cells via the Akt/mTOR pathway. *Molecular Medicine Reports*.

[62] Bi X., Lim J., Henry C. J. (2016). Spices in the management of diabetes mellitus. *Food Chemistry*.

[63] Vanschoonbeek K., Thomassen B. J., Senden J. M., Wodzig W. K., van Loon L. J. (2006). Cinnamon supplementation does not improve glycemic control in postmenopausal type 2 diabetes patients. *Journal of Nutrition*.

[64] Khan A., Bryden N. A., Polansky M. M., Anderson R. A. (1990). Insulin potentiating factor and chromium content of selected foods and spices. *Biological Trace Element Research*.

[65] Zhang Y. F., Chen Y. M., Li L., Hölscher C. (2015). Neuroprotective effects of (Val8)GLP-1-Glu-PAL in the MPTP Parkinson’s disease mouse model. *Behavioural Brain Research*.

[66] Imparl-Radosevich J., Deas S., Polansky M. M. (1998). Regulation of PTP-1 and insulin receptor kinase by fractions from cinnamon: implications for cinnamon regulation of insulin signalling. *Hormone Research*.

[67] Broadhurst C. L., Polansky M. M., Anderson R. A. (2000). Insulin-like biological activity of culinary and medicinal plant aqueous extracts in vitro. *Journal of Agricultural & Food Chemistry*.

[68] Qin B., Nagasaki M., Ren M., Bajotto G., Oshida Y., Sato Y. (2004). Cinnamon extract prevents the insulin resistance induced by a high-fructose diet. *Hormone & Metabolic Research*.

[69] Kim M. K., Hyun S. H., Choung S. Y. (2006). Effect of herbal extract mixtures on serum and liver lipid metabolism in chronic ethanol - administered rats. *Journal of Health Science*.

[70] Babu P. S., Prabuseenivasan S., Ignacimuthu S. (2007). Cinnamaldehyde—a potential antidiabetic agent. *Phytomedicine International Journal of Phytotherapy & Phytopharmacology*.

[71] Jia Q., Liu X., Wu X. (2009). Hypoglycemic activity of a polyphenolic oligomer-rich extract of Cinnamomum parthenoxylon bark in normal and streptozotocin-induced diabetic rats. *Phytomedicine International Journal of Phytotherapy & Phytopharmacology*.

[72] Talpur N., Echard B., Ingram C., Bagchi D., Preuss H. (2005). Effects of a novel formulation of essential oils on glucose-insulin metabolism in diabetic and hypertensive rats: a pilot study. *Diabetes Obesity & Metabolism*.

[73] Khan A., Safdar M., Ali Khan M. M., Khattak K. N., Anderson R. A. (2004). Cinnamon improves glucose and lipids of people with type 2 diabetes. *Diabetes Care*.

[74] Blevins S. M., Leyva M. J., Brown J., Wright J., Scofield R. H., Aston C. E. (2007). Effect of cinnamon on glucose and lipid levels in non insulin-dependent type 2 diabetes. *Diabetes Care*.

[75] Blevins S. M., Leyva M. J., Brown J., Wright J., Scofield R. H., Aston C. E. (2007). Effect of cinnamon on glucose and lipid levels in non insulin-dependent type 2 diabetes. *Diabetes Care*.

[76] Lu T., Sheng H., Wu J., Cheng Y., Zhu J., Chen Y. (2012). Cinnamon extract improves fasting blood glucose and glycosylated hemoglobin level in Chinese patients with type 2 diabetes. *Nutrition Research*.

[77] Ziegenfuss T. N., Hofheins J. E., Mendel R. W., Landis J., Anderson R. A. (2006). Effects of a water-soluble cinnamon extract on body composition and features of the metabolic syndrome in pre-diabetic men and women. *Journal of the International Society of Sports Nutrition*.

[78] Mang B., Wolters M., Schmitt B. (2006). Effects of a cinnamon extract on plasma glucose, HbA 1c, and serum lipids in diabetes mellitus type 2. *European Journal of Clinical Investigation*.

[79] Crawford P. (2009). Effectiveness of cinnamon for lowering hemoglobin A1C in patients with type 2 diabetes: a randomized, controlled trial. *Journal of the American Board of Family Medicine*.

[80] Zhang S., Guohui M. A. (2003). Evaluation of clinical efficacy and review on progress of the thiazolidinedione derivatives (insulin sensitizer). *Evaluation and Analysis of Drug-use in Hospital of China*.

[81] Lachmann M. J., Salgin B., Kummer S. (2016). Remission of congenital hyperinsulinism following conservative treatment: an exploratory study in patients with KATP channel mutations. *Journal of Pediatric Endocrinology and Metabolism*.

[82] Gwinn D. M., Shackelford D. B., Egan D. F. (2008). AMPK phosphorylation of raptor mediates a metabolic checkpoint. *Molecular Cell*.

[83] Fajas L. (1998). A Pro12Ala substitution in PPARγ2 associated with decreased receptor activity, lower body mass index and improved insulin sensitivity. *Nature Genetics*.

[84] Wu T., Xie C., Wu H., Jones K. L., Horowitz M., Rayner C. K. (2017). Metformin reduces the rate of small intestinal glucose absorption in type 2 diabetes. *Diabetes Obesity & Metabolism*.

[85] Toda C., Kim J. D., Impellizzeri D., Cuzzocrea S., Liu Z. W., Diano S. (2016). UCP2 regulates mitochondrial fission and ventromedial nucleus control of glucose responsiveness. *Cell*.

[86] Dooley J., Tian L., Schonefeldt S. (2016). Genetic predisposition for beta cell fragility underlies type 1 and type 2 diabetes. *Nature Genetics*.

[87] Scarlett J. M., Rojas J. M., Matsen M. E. (2016). Central injection of fibroblast growth factor 1 induces sustained remission of diabetic hyperglycemia in rodents. *Nature Medicine*.

[88] Bader E., Migliorini A., Gegg M. (2016). Identification of proliferative and mature *β*-cells in the islets of Langerhans. *Nature*.

[89] Wei X., Song H., Yin L. (2016). Fatty acid synthesis configures the plasma membrane for inflammation in diabetes. *Nature*.

[90] Li P., Liu S., Lu M. (2016). Hematopoietic-derived galectin-3 causes cellular and systemic insulin resistance. *Cell*.

[91] Cadario F., Savastio S., Rizzo A. M., Carrera D., Bona G., Ricordi C. (2017). Can type 1 diabetes progression be halted? Possible role of high dose vitamin D and omega 3 fatty acids. *European Review for Medical & Pharmacological Sciences*.

[92] Chen Z., Sun M., Yuan Q. (2016). Generation of functional hepatocytes from human spermatogonial stem cells. *Oncotarget*.

[93] Eldin I., Ahmed E. M., Ae H. M. (2009). Preliminary study of the clinical hypoglycemic effects of *Allium cepa* (red onion) in type 1 and type 2 diabetic patients. *Environmental Health Insights*.

[94] Anderson R. (2006). Cloves improve glucose, cholesterol and triglycerides of people with type 2 diabetes mellitus. *The FASEB Journal*.

[95] Li Z., Henning S. M., Zhang Y. (2013). Decrease of postprandial endothelial dysfunction by spice mix added to high-fat hamburger meat in men with type2 diabetes mellitus. *Diabetic Medicine: A Journal of the British Diabetic Association*.

[96] Li Y., Tran V. H., Duke C. C., Roufogalis B. D. (2012). Gingerols of *Zingiber officinale* enhance glucose uptake by increasing cell surface GLUT4 in cultured L6 myotubes. *Planta Medica*.

